# 1559. Healthcare Staff Perceptions of Feasibility and Acceptability on Implementing Injectable HIV Pre-exposure Prophylaxis into Standard of Care: Baseline Results from the PrEP Implementation Study for Cabotegravir Long Acting for Men in the Real World (PILLAR)

**DOI:** 10.1093/ofid/ofad500.1394

**Published:** 2023-11-27

**Authors:** Julian A Torres, Dima Dandachi, Hadrian Holder, Bo Li, Alison Gaudion, Deanna Merrill, David Andrae, William Lenderking, Riya Moodley, Annemiek de Ruiter, Maggie Czarnogorski, Nanlesta Pilgrim

**Affiliations:** Montefiore Medical Center, Albert Einstein College of Medicine, Bronx, New York; University of Missouri - Columbia, Columbia, Missouri; Southwest Community Health Center, Bridgeport, Connecticut; GSK, Collegeville, Pennsylvania; ViiV Healthcare, Brentford, England, United Kingdom; ViiV Healthcare, Brentford, England, United Kingdom; Evidera Inc., Bethesda, Maryland; Evidera Inc., Bethesda, Maryland; ViiV Healthcare, Brentford, England, United Kingdom; ViiV Healthcare., London, England, United Kingdom; ViiV Healthcare, Brentford, England, United Kingdom; ViiV Healthcare, Brentford, England, United Kingdom

## Abstract

**Background:**

PILLAR evaluates the feasibility and acceptability of implementation strategies for delivering long-acting Cabotegravir for PrEP (CAB LA) to men who have sex with men and transgender men in low and high-volume PrEP Sites across the United States. We report staff study participants’ (SSPs) baseline perceptions of implementation prior to study sites commencing enrollment and using implementation strategies.

**Methods:**

86 SSPs from 17 clinics completed surveys on implementation outcomes assessed using the acceptability of intervention measure (AIM) and feasibility of intervention measure (FIM), whose summary scores are averages of four items measured on a 5-point rating scale (1=completely disagree to 5=completely agree). Perceived barriers to CAB LA implementation were assessed via a 5-point rating scale (1=extremely concerned to 5=not at all concerned). Results were compared by clinic volume of PrEP seeking individuals where high-volume sites (HVS) were defined as serving greater than 50 persons per month.

**Results:**

Table 1 reports SSP demographics. SSPs reported high levels of feasibility and acceptability of implementing CAB LA (mean FIM=4.4 and mean AIM=4.7) and implementation support (mean FIM=4.1 and mean AIM=4.0) (Table 2). SSPs at low volume sites’ (LVS) had higher levels of feasibility and were more extremely positive about implementing CAB LA (71% vs. 34% ) compared to those at HVS.

Top perceived barriers to delivering CAB LA, “moderately concerned” to “extremely concerned” SSP ratings included: medication cost (51%), patients’ ability to keep appointments (32%), patients’ willingness to travel for 2-monthly appointments (28%), ability to identify and flag missed injection visits (23%), and staff resourcing (20%) (Table 3). A higher proportion of HVS’ SSPs reported being concerned about these barriers. SSPs were least concerned about the gluteal medial injection, managing oral lead-in, the medication’s efficacy, and patients feeling stigmatized.

**Figure:**
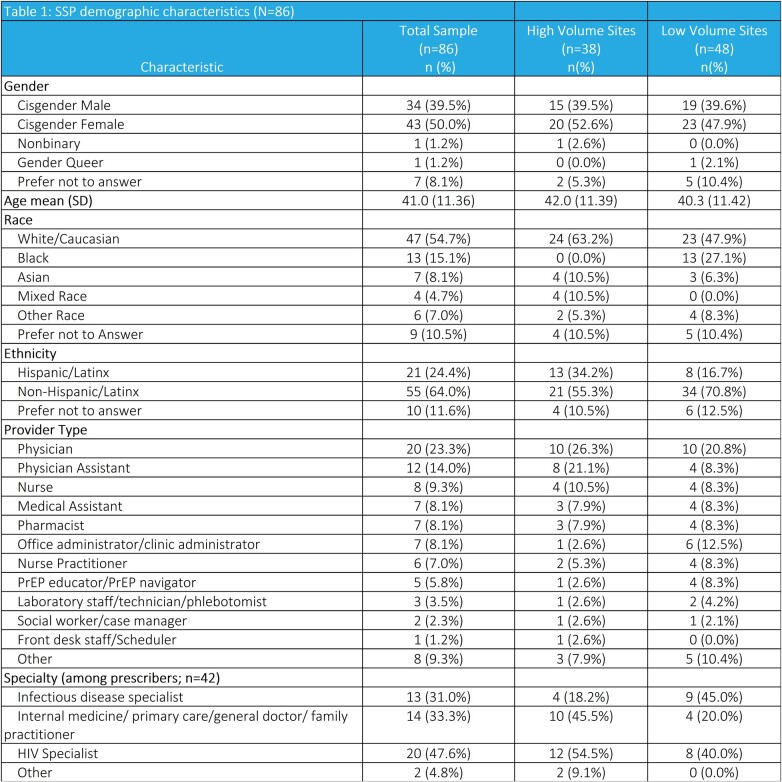

**Figure:**
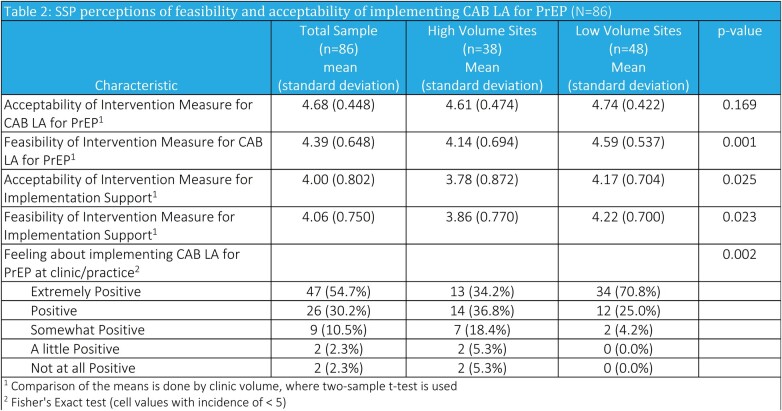

**Figure:**
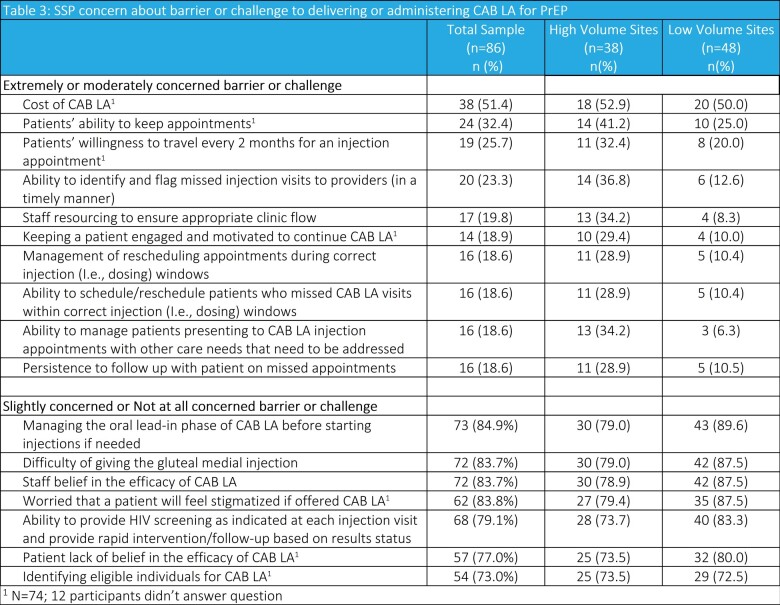

**Conclusion:**

At baseline, SSPs found CAB LA to be highly acceptable and feasible to implement into standard of care with notable differences by site volume. HVS may be more aware of PrEP introduction complexities than LVS. Support with benefits navigation, scheduling and managing missed injection visits is key for SSPs.

**Disclosures:**

**Dima Dandachi, MD, MPH**, ViiV Healthcare: Advisor/Consultant|ViiV Healthcare: Grant/Research Support **Bo Li, PhD**, GSK: Employment|GSK: Stocks/Bonds **Alison Gaudion, PhD**, ViiV Healthcare: Employment|ViiV Healthcare: Stocks/Bonds **Deanna Merrill, PharmD, MBA, AAHIVP**, ViiV Healthcare: Employment|ViiV Healthcare: Stocks/Bonds **David Andrae, PhD**, Evidera: Employment **William Lenderking, PhD**, Evidera: Employment|Pfizer: Former Employment|Pfizer: Stocks/Bonds **Riya Moodley, FCP**, ViiV Healthcare: Employment|ViiV Healthcare: Stocks/Bonds **Annemiek de Ruiter, MBBS FRCP**, ViiV Healthcare: Employment|ViiV Healthcare: Stocks/Bonds **Maggie Czarnogorski, MD MPH**, ViiV Healthcare: Employment|ViiV Healthcare: Stocks/Bonds **Nanlesta Pilgrim, PhD**, ViiV Healthcare: Employment|ViiV Healthcare: Stocks/Bonds

